# Engineering immune-competent niches: strategies, applications, and translational hurdles in ovarian cancer organoid models

**DOI:** 10.3389/fimmu.2026.1773734

**Published:** 2026-03-12

**Authors:** Yuying Chen, Na Xu, Mingxin Dong, Wensen Liu, Ziwei Liu, Guangchao Sun, Yan Jia

**Affiliations:** 1 Department of Gynecology and Obstetrics, The Second Hospital of Jilin University, Changchun, Jilin, China; 2 Academic Affairs Office, Jilin Medical University, Jilin, Jilin, China; 3 Changchun Veterinary Research Institute, Chinese Academy of Agricultural Science, Changchun, Jilin, China; 4 Department of General Surgery, The Second Hospital of Jilin University, Changchun, Jilin, China

**Keywords:** 3D culture, immunotherapy, ovarian cancer, tumor organoids, tumor microenvironment

## Abstract

Ovarian cancer remains the most lethal gynecologic malignancy due to strong interpatient heterogeneity and immune evasion. Traditional two-dimensional cultures and animal models lack the ability to maintain interactions among tumors, immune cells, and stromal cells and have limitations in clinical translation. This review discusses the organoid construction methods using adult stem cells (normal epithelium, tumor tissues and ascites), and induced pluripotent stem cells, comparing various culture platforms from air–liquid interface to microfluidic devices. We highlight organoids containing immune components are valuable for assessing T cell exhaustion, NK cell cytotoxicity, and stromal communication, which help to screen immunotherapy, discover biomarker, and profile drug resistance. The persistent challenges include limited vascularization, short-term maintenance of immune components and lack of standard protocols. We present new solutions that integrate multi-omics, biomaterials and automated perfusion to improve physiological fidelity and scalability. Collectively, ovarian cancer organoids with immune microenvironment can bridge preclinical gaps and accelerate the development of personalized immune therapy.

## 1 Introduction

Ovarian cancer (OC) is the most lethal gynecologic malignancy, accounting for more than 300,000 new diagnoses and over 200,000 deaths worldwide each year, with five-year survival rates below 50% in most advanced-stage patients (1). Despite the advent of Poly Adenosine Diphosphate-ribose Polymerase (PARP) inhibitors and immune checkpoint inhibitors (ICIs), therapeutic gains remain modest because high-grade serous ovarian carcinoma (HGSOC) exhibit profound intra- and interpatient heterogeneity, rapid evolution of chemoresistance, and an immune landscape dominated by dysfunctional T cells, immunosuppressive myeloid populations, and desmoplastic stroma (2–5). Recent studies have further elucidated the complex molecular landscapes and pathological mechanisms driving tumorigenesis and therapeutic failure (6–9). Conventional two-dimensional (2D) cultures and patient-derived xenograft (PDX) models incompletely preserve the tumor microenvironment (TME), often losing spatial organization, stromal cues, and native immune repertoires, thereby limiting their utility for mechanistic discovery or immunotherapy screening (10–12). (**Table 1**) Consequently, there is an urgent need for preclinical models that can faithfully recapitulate these biological complexities to facilitate the development of novel therapeutic strategies.

**Table 1:** Summary of preclinical models for cancer research.

Model	Advantages	Disadvantages
2D Cell Line Models	Simple operation, low cost, high throughput, rapid expansion	Poor recapitulation of complex tumor biology, potential for genetic drift
PDX Models	Patient-specific, preserves tumor heterogeneity and architecture	Lacks a functional immune system, host stromal replacement, variable success rate, high cost
Genetically Engineered Mouse Models	Intact immune system, suitable for studying carcinogenesis mechanisms and mechanisms of *in situ* tumor formation, possesses a complete TME	High cost, long modeling time, technically challenging, lacks human-specific context
Tumor Spheroid Models	Technically simple, high throughput, relatively low cost	Relatively simple structure, lacks TME components, limited maintenance time
Tumor Tissue Explants	Contains tumor cells, stromal and immune cells, preserves tumor architecture, simulates TME	TME characteristics can only be maintained short-term, presents technical challenges
Epithelial Tumor Organoid Models	Retains tumor heterogeneity, patient-specific, suitable for high-throughput screening	Lack of standardized establishment protocols, lacks a complete TME, relatively high cost
Immune-Competent Organoids	Capable of modeling tumor-immune interactions, high throughput, tunable ratios of immune and tumor cells	Incomplete TME representation, technically complex, time-consuming, costly, limited reproducibility

In this review, we first outline the clinical need to recapitulate tumor–immune–stromal interactions, then survey emerging OC organoid platforms derived from adult stem cells (ASCs) and induced pluripotent stem cells (iPSCs) (13). Particular emphasis is placed on how these systems model immune evasion, support co-cultures with T cells, NK cells, and antigen-presenting cells, and enable preclinical testing of checkpoint blockade, adoptive cell therapies, and bispecific antibodies (14, 15). We also address key bottlenecks such as immune cell persistence, vascularization, and microbial influences (16). Our goal is to design immune-competent organoid platforms that can accelerate personalized immuno-oncology and improve clinical outcomes for OC patients.

## 2 Establishment of tumor organoids

OC organoids rely on diverse cellular sources to model tumorigenesis and the TME. This section outlines the characteristics and limitations of the two primary cell sources, followed by an evaluation of organoid utility across specific histological subtypes, including HGSOC, ovarian clear cell carcinoma (OCCC), endometrioid carcinoma (EnOC), and mucinous carcinoma (MC).

### 2.1 Sources of tumor organoids

**Figure 1:**
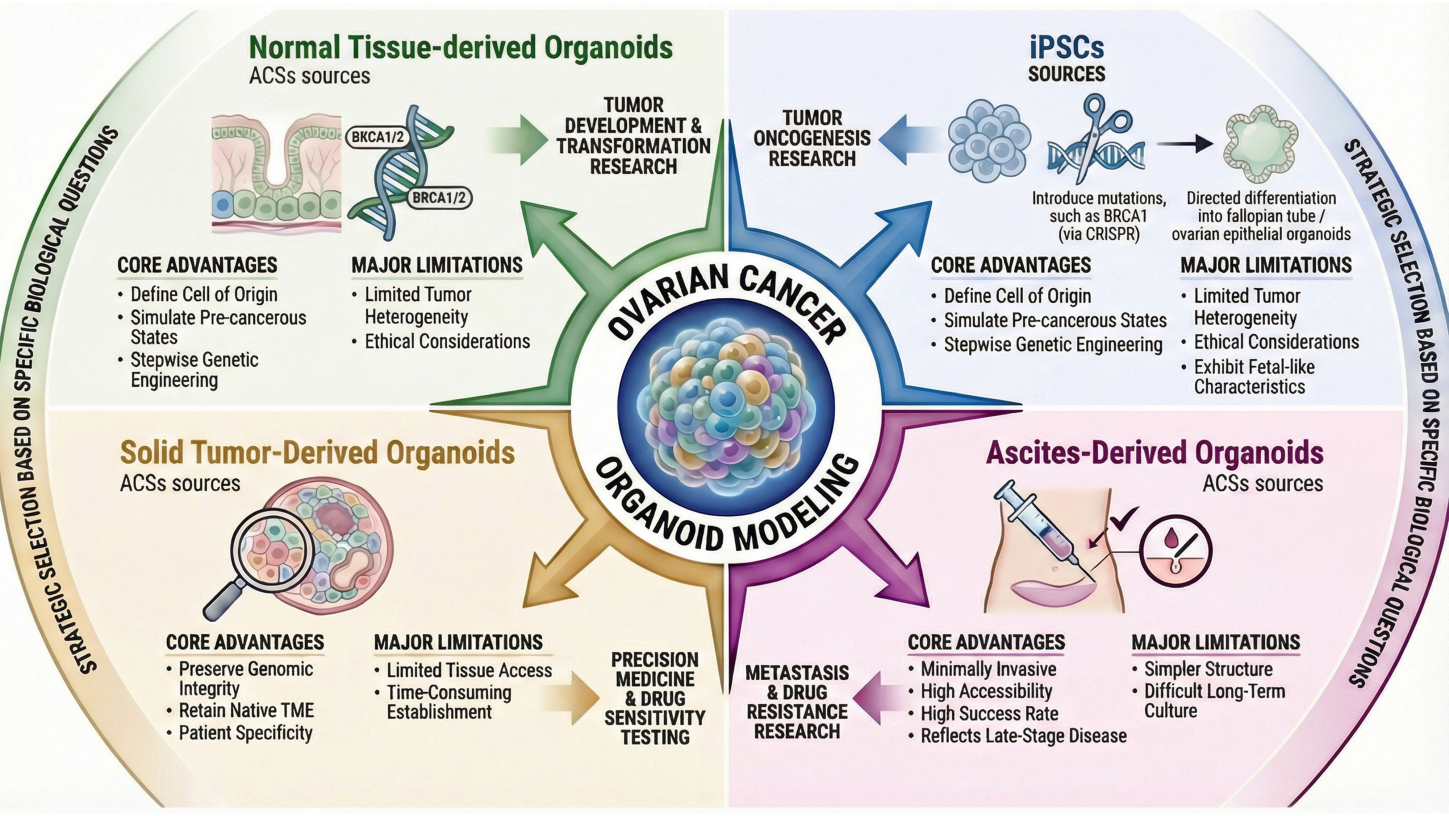
Strategic landscape of OC organoid sources. This diagram systematically compares the two primary cellular sources for deriving OC organoids and highlights their distinct advantages, limitations, and optimal applications.

The development of OC organoid models is primarily based on two main cellular sources (13, 17): iPSCs and ASCs. While iPSCs are reprogrammed from somatic cells, ASCs can be enriched in culture from primary tissues, including healthy epithelium for modeling tumor origin and, in the case of cancer organoids, tumor tissue or malignant ascites. Each source presents distinct advantages and limitations, thereby providing a versatile toolkit for mechanistic studies and personalized therapy (**Figure 1**).

#### 2.1.1 iPSCs-derived organoids

IPSCs provide a platform for studying the mechanisms of early tumorigenesis and enable precise introduction of pathogenic mutations through gene editing into tissues. Yucer et al. (18) established a protocol to differentiate human iPSCs into functional fallopian tube (FT) epithelial organoids. In the following study, utilizing iPSCs derived from germline BRCA1 mutation carriers, they produced a model that recapitulates early carcinogenesis of hereditary OC (19). IPSC-derived organoids are useful for functional genomics and high-throughput screening (20), but they lack an authentic TME and often exhibit fetal-like characteristics rather than fully mature adult tissue phenotypes (21). Furthermore, their application is constrained by technical challenges related to the complexity and variability of directed differentiation protocols (22).

#### 2.1.2 ASCs-derived organoids

ASCs reside in specific tissues and can be isolated to generate organoid models. In the context of OC research, ASCs-derived models are generally categorized based on the disease state of the source tissue: normal tissue-derived organoids (for modeling tumor initiation) and patient-derived organoids (PDOs) (derived from solid tumors or ascites for personalized medicine).

##### 2.1.2.1 Normal tissue-derived organoids

ASCs isolated from non-malignant tissues provide an alternative approach to model tumor development, especially cell origin. The genetic engineering of ASCs facilitates the stepwise introduction of oncogenic mutations. Kopper et al. (23) derived organoids from normal FT and ovarian surface epithelium (OSE) of BRCA1/2 mutation carriers. Interestingly, organoids from high-risk individuals clustered with cancer organoids, suggesting a pre-neoplastic state, which can lead to malignant transformation. Zhang et al. (24) utilized gene-edited mouse models to demonstrate that both FT and OSE can be origins for HGSOC, and show different biological pathways. However, engineered normal ASCs models may not fully capture the complex genetic heterogeneity of patient tumors and may involve ethical considerations in tissue acquisition (25, 26).

##### 2.1.2.2 Solid tumor-derived organoids

PDOs derived from tumor tissue have been shown to faithfully preserve the genomic integrity of the original tumor. Furthermore, they retain the native TME, particularly during the first stages of *in vitro* culture (27).For example, Mercadante et al. (28) developed an organoid model using fresh OC tissues, which retained patient tumor heterogeneity and captured intratumor response heterogeneity to NK cell cytotoxicity. Furthermore, Cai et al. (29) generated organoids that recapitulated key features of TME, providing a robust platform for drug sensitivity testing and molecular profiling. A main limitation of this approach is the restricted availability of fresh tumor tissue and the time-consuming in the establishment and expansion of organoids (30).

##### 2.1.2.3 Ascites-derived organoids

Malignant ascites offers a liquid biopsy source for ASCs, providing a minimally invasive and efficient method with high success rates (31, 32). This approach is particularly useful for studying metastasis and drug resistance in advanced tumors. Wu et al. (31) optimized protocols using three-dimensional (3D) matrigel culture and defined media to obtain ascites-derived organoids which retained patient-specific features and facilitated the modeling of platinum resistance. Thorel et al. (32) further demonstrated that ascites-derived organoids preserve key cell features of both primary and metastatic tumors, enabling the serial monitoring of therapeutic sensitivity. However, these models often exhibit simpler structures compared to tissue-derived organoids and can be difficult to sustain in the long-term culture (33). In conclusion, the selection of the organoid source depends on the specific biological question: iPSCs and normal ASCs are ideal for studying early carcinogenesis and cell of origin, whereas PDOs (derived from either solid tissue or ascites) are indispensable for studying established disease heterogeneity, TME interactions, and personalized therapeutic responses.

### 2.2 Subtype-specificity in OC organoid models

HGSOC is the most common type of OC pathology, but other histological subtypes like OCCC, EnOC and MC display different molecular features and responses to chemotherapy.

Recent studies have highlighted the effectiveness of organoid technology in modeling these subtypes. In contrast to HGSOC, which relies on particular microenvironments (e.g., low Wnt environment (34)) to inhibit the proliferation of normal epithelial cells, non-HGSOC subtypes generally have higher initiation rates because of their distinct driver mutations. Kopper et al. (23) have developed a biobank comprising OCCC, EnOC, and MC cell lines, illustrating that OCCC and EnOC organoids can faithfully retain important mutations such as ARID1A and PIK3CA, which are less common in HGSOC. Furthermore, organoids derived from OCCC have proven particularly valuable for investigating mechanisms of platinum resistance, a clinical hallmark of this subtype. Results show that OCCC organoids can faithfully recapitulate the chemo-resistant phenotype of patients and can be used to screen targeted therapies such as EZH2 inhibitors that may not be found in HGSOC alone (23, 35). Different culture protocols have been optimized for MC, which requires specific growth factors (e.g. Neuregulin-1) to maintain the amplification of ERBB2 or KRAS mutations typical of this subtype (23). These results show that patient-derived organoids can capture the heterogeneity of the entire spectrum of OC, and are of use outside HGSOC to other subtypes.

## 3 Culture approaches and strategies for immune-competent organoids

**Figure 2:**
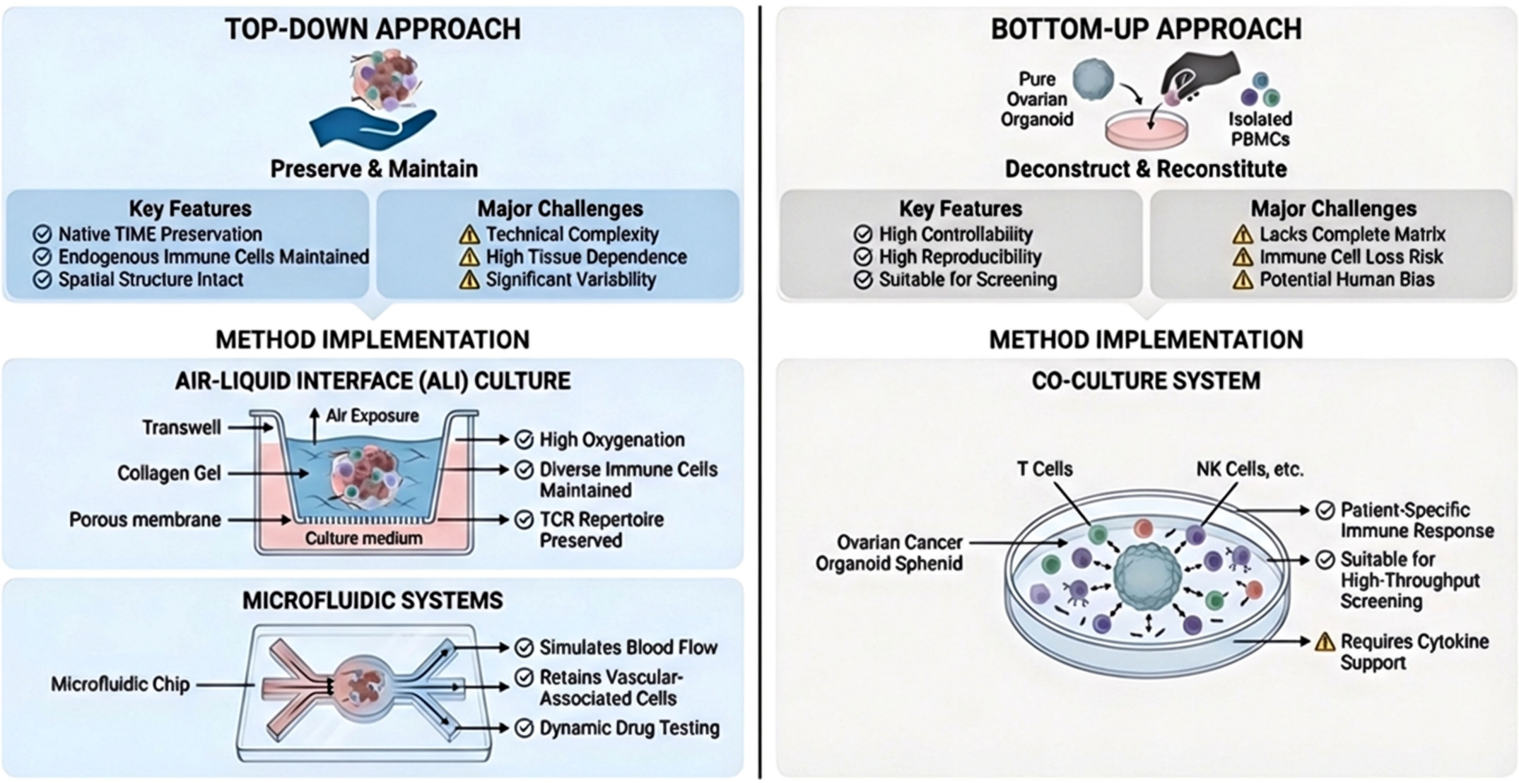
Strategic comparison for building immune-active OC organoids. This schematic contrasts the top-down approach with the bottom-up approach, along with their key methods. This framework guides the selection of a strategy by balancing the need for physiological fidelity against the requirements for experimental control and scalability in immunotherapy research.

To recapitulate the complex interaction between OC and the immune system, researchers developed various *in vitro* platforms, such as holistic and reductionist, as well as culture methods including co-culture systems, air–liquid interface (ALI) cultures, and microfluidic platforms (**Figure 2**).

### 3.1 Construction strategies: top-down vs. bottom-up approaches

The design of immune-competent organoids generally follows one of two strategies:

The top-down approach aims to preserve the native cellular composition of the original tumor. This method involves mechanically mincing fresh tumor tissue into micro-fragments and culturing them directly in ALI or microfluidic systems (36). The “whole-tumor” organoids retain epithelial cells, endogenous immune cells and stromal components. This strategy maintains the native tumor immune microenvironment and facilitates *in situ* survival and activation of tumor-specific T cells, especially when supplemented with cytokines like Interleukin (IL)-2 (37). Due to their faithful preservation of parental tumor including cells and spatial structure, top-down models offer high physiological relevance for studying endogenous immune responses.

In contrast, the bottom-up approach utilizes a stepwise construction process involving tumor epithelial cells, immune cells or stromal cells. Tumor epithelial organoids are initially isolated and expanded as pure cultures, then reconstituted *in vitro* with autologous cells(e.g., peripheral blood mononuclear cells or defined T cell subsets) from the same patient (36). The strength of this method lies in its reproducibility and controllability. It supports the long-term expansion of tumor epithelial cells and permits precise manipulation of immune components, making it a practical platform for mechanistic investigation and high-throughput drug screening. To enhance complexity, composite systems can be developed by reintroducing stromal cells alongside immune cells, enabling the simulation of dynamic processes like immune cell infiltration and cytotoxicity (38).

### 3.2 Key culture methods

Traditional organoid cultures lack stromal and immune components, which limits their utility for studying immunotherapy. To address this, several advanced culture strategies have been developed to preserve or reconstitute the native TME. These methods can be classified based on their engineering approach: bottom-up reconstruction versus top-down preservation.

Co-culture Systems (Bottom-up Approach): Organotypic co-culture is widely used to generate immune-competent models. Tumor organoids are embedded in a basement membrane extract matrix and cultured with immune cells in an enriched medium (39, 40). This arrangement facilitates the expansion of tumor-reactive T cells and can recapitulate patient-specific immune responses (39). Recent advancements include co-culture with resident immune cells of tumors; for instance, intraepithelial lymphocytes were maintained and expanded in mouse intestinal organoids using IL-2, IL-7, and IL-15 (41). However, a main limitation of this approach is the gradual loss of immune cells over time due to the lack of an optimal shared culture medium, which can equally support the survival of both epithelial and immune cells (42).

ALI Cultures (Top-down Approach): The ALI method represents a top-down approach that preserves the endogenous TME by embedding intact tumor fragments in a collagen gel on a transwell insert, with the apical surface exposed to air (37). This setup ensures adequate oxygenation and preserve the native tumor structure and endogenous immune components (37, 43). ALI cultures can sustain various immune cell types including macrophages, cytotoxic T cells, B cells, NK cells and native T cells, for up to 30 days (37). This demonstrated the platform effectively models immune checkpoint blockade, activating tumor antigen specific T cells. Although its high biomimicry, ALI cultures can be technically challenging and may exhibit variability inherent to the primary tumor tissue.

Microfluidic Systems (Top-down Approach): Microfluidic chips cultivate organoids in a dynamic fluid environment mimicking *in vivo* flow and nutrients. Tissue fragments are loaded into microchannels and interact with epithelial, stromal, and immune cells under physiological flow conditions (44). This is particularly relevant for OC, which primarily spreads through ascites dissemination under constant fluid shear forces. Systems with continuous fluid circulation can replicate the hydrodynamic conditions of the abdominal cavity (45). The application of shear force has been shown to boost the epithelial-mesenchymal transition of tumor cells, facilitating their attachment to mesenchymal cells (46, 47). Futhermore, mouse-derived and patient-derived organotypic tumor spheroids have shown that these systems can retain autologous hematopoietic cells and replicate clinical responses to programmed cell death protein (PD)-1 blockade (44, 48). Although powerful for simulating dynamic flow conditions, microfluidic systems require special equipment and face challenges related to throughput and standardization (49).

Scaffold-free Suspension Models: To model specific architecture of malignant ascites, stent-free culture methods (e.g., generating “tumor spheroids” using magnetic levitation (50, 51) or suspension drop (52))must be applied. The extracellular matrix is removed and cells form dense aggregates through intrinsic interactions (53). This model closely reproduces typical cell structure in OC ascites and it provides a platform for experimental studies of anti-metastatic drug efficacy.

Persistence of endogenous immune cells is different across platforms, and this tradeoff can be made between physiological fidelity and experimental scaleability. In co-culture systems, immune populations are rapidly depleted within 7–14 days due to lack of stromal support (39). Top-down models such as ALI method and microfluidics exploit preserved native stroma or dynamic fluid flow to extend immune cell viability and functionality to 28–60 days (37) and 3–4 week (44) respectively. However, these high-fidelity systems are technically demanding and limited by tissue availability. In contrast, bottom-up co-culture systems offer better controllability and reproducibility but lack the full stromal complexity of native tumor. Ultimately, model selection must balance long-term immune maintenance and experimental reproducibility.

### 3.3 Strategic framework for model selection

Selecting the appropriate organoid culture method is critical and should be dictated by the specific research objective (**Table 2**).

**Table 2:** Strategic selection of OC organoid models based on research objectives.

Research goal	Recommended models	Advantages
Large-scale Drug Screening/Precision Medicine	ECM-embedded PDOs	High scalability, reproducibility
Immuno-Oncology/TME	ALI/Co-culture	Preservation of immune cells
Metastasis/Vascularization	Microfluidics	Dynamic flow, tissue interfaces
Early Carcinogenesis	iPSC-derived/Gene-edited ASCs	Stepwise modeling of “Cell of Origin”

For high-throughput drug screening and precision medicine: Standard tumor-derived organoid model (often established in extracellular matrix (ECM) domes) remains the preferred choice. Its simplicity, scaleability, and high expansion efficiency make it the optimal choice for large-scale compound screening and living biobanks, where experimental reproducibility is paramount.

For immuno-oncology and TME studies: When the goal is to evaluate immunotherapies or study tumor-stroma interactions, ALI or co-culture systems are recommended. These methods can be used to preserve native immune cells and stromal components lost in standard cultures.

For metastasis and vascularization mechanisms: Cancer cell invasion, metastasis, or shear stress responses benefit most from microfluidic platforms. These systems capture dynamic blood flow and complex tissue interfaces.

For early carcinogenesis and prevention: To study cell origin or stepwise accumulation of mutations, iPSC-derived models or CRISPR-edited normal ASCs are preferred, because they allow for precise modeling of pre-neoplastic states from a clean genetic background.

## 4 Advancements and innovations in construction strategies and multimodal analysis

**Figure 3:**
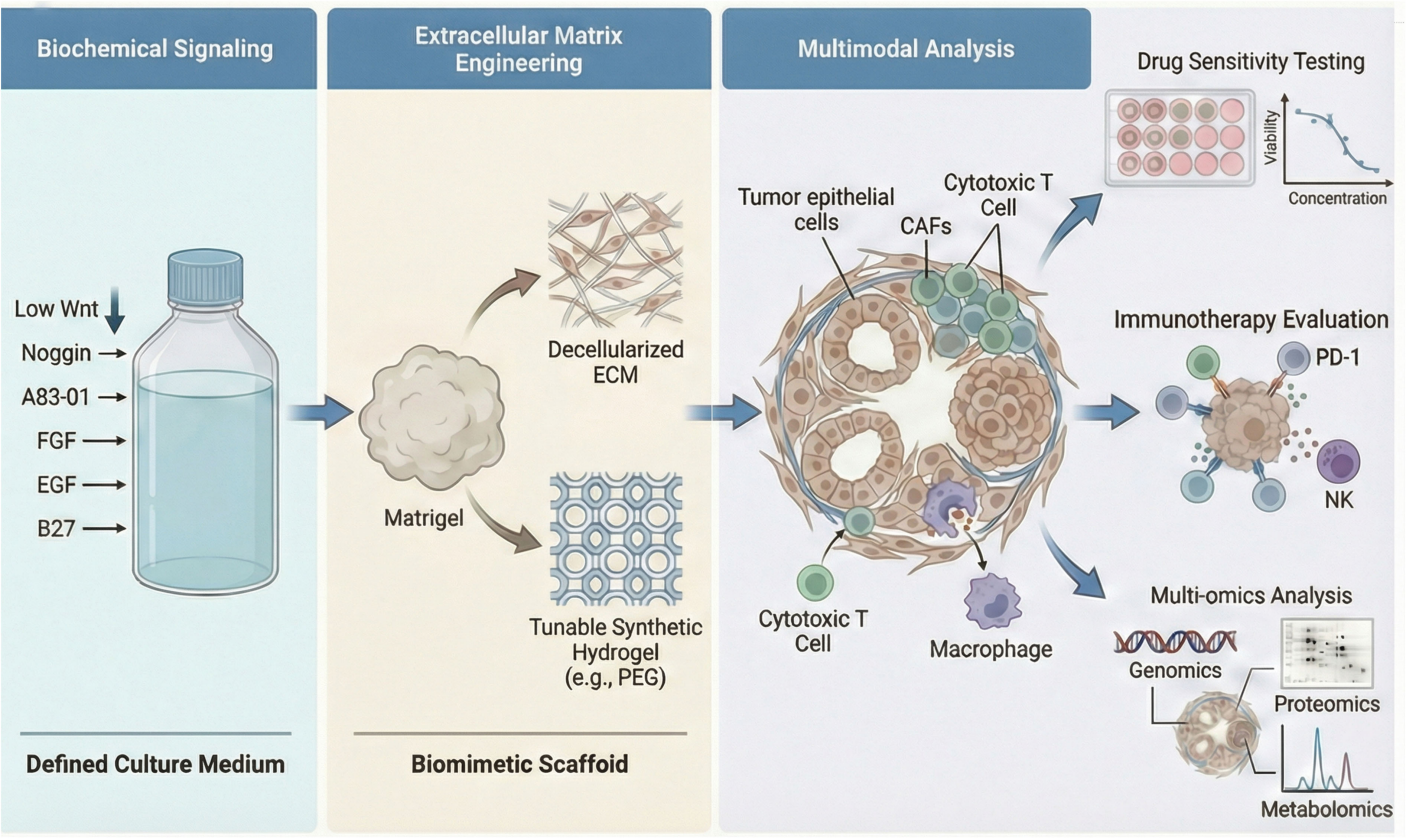
Integrated technological platform for constructing high-fidelity OC organoids. A unified platform for creating realistic OC models. This diagram shows how combining tailored growth conditions, advanced biological scaffolds, and detailed analysis techniques produces organoids that can accurately test drugs and new immunotherapies.

The generation of high-fidelity OC organoids is a complex process, with success rates varying from 55% to 100% (23, 28, 54–60). This efficiency is influenced by multiple factors, including the combinations of growth factors, the type of extracellular matrix, the quality of the tumor sample, the initial cell number, and the presence of stromal and immune cells in the microenvironment (**Figure 3**).

### 4.1 Establishment and optimization of culture conditions

The framework for organoid culture is established using a universal medium based on Advanced DMEM/F12, supplemented with HEPES, GlutaMAX, and B27 (23). However, the success rate of organoid construction and the long-term amplification capacity of OC, particularly HGSOC, are highly dependent on the precise regulation of key signaling pathways.

In most epithelial organoid cultures, such as the intestine, exogenous Wnt3a is crucial for maintaining homeostasis (61). However, HGSOC organoids exhibit distinct regulation of Wnt signaling. Kopper et al. (23) highlighted in their seminal work that HGSOC organoids not only do not depend on exogenous Wnt3a but also exhibit a detrimental reaction to elevated levels of Wnt signals. This response may stem from the autonomous activation or downstream mutations in the Wnt pathway within HGSC cells. Excessive exogenous Wnt activation can overwhelm the signaling cascade, leading to cellular senescence or growth arrest (34). Notably, normal FT epithelium, the primary source of HGSOC, heavily relies on Wnt3a for growth (62). Therefore, this fundamental difference in Wnt dependency between normal FT epithelium and HGSOC organoids provides a critical theoretical basis for selectively enriching tumor cells by modulating Wnt levels in culture media. The inhibition of the TGF-β superfamily is essential for preserving an undifferentiated state and preventing epithelial-mesenchymal transition in organoids. Noggin, an antagonist of bone morphogenetic protein(BMP), is a standard component of HGSOC medium. It effectively inhibits the differentiation process induced by BMP signals, thereby sustaining the proliferative capacity of tumor cells (23). A83-01, an inhibitor of activin receptor-like kinase4/5/7, blocks the TGF-β signaling pathway. Research indicates that in the absence of A83-01, organoids are prone to enter a stagnation phase after several passages due to telomere attrition or stress responses. This compound works synergistically with Noggin, significantly extending the *in vitro* lifespan of organoids (23, 34).

In addition to fundamental survival signals, specific growth factors and hormones are essential for mimicking the *in vivo* microenvironment (63).To optimize the establishment efficiency of HGSOC organoids, Senkowski et al. developed a defined medium supplemented with key mitogens, including fibroblast growth factor (FGF)-4 and FGF-10, which supported robust long-term expansion while excluding Wnt to prevent normal tissue overgrowth (54). FGF signaling pathway promotes chemoresistance in OC by regulating the ability of homologous recombination-mediated DNA damage repair (64). Furthermore, high concentrations of nicotinamide, as precursors of ROCK kinase modulators and PARP inhibitors, inhibit cell differentiation and facilitate the formation of dense cystic structures in organoids (27). Due to hormone dependent nature of OC, *β*-estradiol is often added to the culture medium. This supplementation recaptures physiological hormone environment and maintain estrogen receptor activity, thereby aligning the organoid phenotype more closely to the original tumor (23). During the initial establishment of organoids and throughout subsequent passages, cells exhibit a heightened susceptibility to Anoikis. Consequently, the incorporation of Rho kinase inhibitors, such as Y-27632, is essential, as it significantly enhances the survival rate of single cells following dissociation by stabilizing the cytoskeleton (23).

In conclusion, optimizing the culture medium for HGSOC organoids involves balancing the need to “maintain stemness” while preventing differentiation and senescence. A precise formula is essential not only for promoting growth but also for effectively screening tumor cells and minimizing the impact of normal cells.

### 4.2 ECM as a critical scaffold

Although basement membrane extracts such as Matrigel or Cultrex are widely used for organoid culture, their uncertain composition and mouse sarcoma-derived characteristics lead to significant batch variations and potential immunogenicity, hampering their clinical translational prospects (65). The ECM provides structural support, intercellular communication, and mechanical signals. Research indicates that extracellular vesicles released by tumor cells can modify distant ECM microenvironment, leading to enhancements like elevated fibronectin deposition. Consequently, these ECM modifications supply physical and biochemical clues needed for tumor cell formation and proliferation (66). As evidenced bythe frequent spread of HGSOC to collagen-rich, remodeled omental tissue (67). Research indicates that stiff matrices can trigger the YAP/TAZ signaling pathway, prompting the upregulation of chemotherapy resistance-related genes at the transcriptional level and boosting tumor stemness, thereby directly fostering drug resistance (68). Controllable synthetic hydrogels, such as polyethylene glycol-based materials, are increasingly preferred due to their ability to isolate the biochemical cues and mechanical properties of the matrix for investigation (69). Studies have shown that merely replicating high stiffness *in vivo* can lead to platinum resistance in OC. To better mimic the organ-specific *in vitro* environment, researchers are creating acellular ECMs sourced from bovine or human retinas (69). These decellularized ECMs preserve the “matrix memory” of the original tissue, including tissue-specific hidden peptides and growth factor reservoirs essential for maintaining the distinct invasive phenotype of OC cells (70). For clinical translation, synthetic hydrogels (e.g., PEG) have a defined composition and high reproducibility, but they require bioactive functionalization to overcome their inertness (71). Decellularized ECM retains native biochemical information for high-fidelity modeling but is limited by batch variability and complex preparation (72).

### 4.3 Multimodal analysis for high-resolution characterization

The integration of multimodal analysis techniques is transforming organoids from 3D culture containers into intricate biological databases. Consequently, the research focus has shifted from basic endpoint survival rate detection to a comprehensive examination of spatiotemporal heterogeneity and cellular functions. Traditional sequencing often obscures the information pertaining to key rare cell subpopulations (5). In contrast, spatial transcriptomics enables the mapping of the “cell map” within organoids, allowing for the precise identification of resting cell regions induced by hypoxia and the identification of drug-resistant persistent cells located in the core (73). This understanding is essential for elucidating the mechanisms underlying tumor recurrence following treatment (74). Fluorescence lifetime imaging microscopy enables the sensitive detection of early metabolic changes through real-time monitoring of metabolic coenzymes within cells (75). This approach allows for the prediction of a drug’s therapeutic effects several hours post-treatment, significantly preceding the onset of morphological changes like apoptosis (76). The integration of CRISPR-Cas9 gene editing and screening technology with patient-derived organoid platforms has created new opportunities for functional genomics research (14). Researchers can systematically knock out specific genes in organoids, facilitating the identification of potential therapeutic targets within the context of patient-specific genetic backgrounds (77).

## 5 Applications

### 5.1 Dissecting the immunosuppressive microenvironment in OC

While traditional models face limitations in elucidating mechanisms of tumor invasion and drug resistance (78), organoid platforms offer a powerful tool to unravel the multi-layer immune evasion mechanisms in OC. HGSOC organoids remodel the immune landscape by secreting cytokines. Blocking C-X-C motif chemokine ligand 10 reduces T-cell infiltration, and neutralizing granulocyte-macrophage colony-stimulating factor reduces the number of tumor-associated macrophages and myeloid suppressor cells (14). In this suppressive microenvironment, defects in the tumor cells themselves are key drivers of escape. Disrupting direct the antigen presentation machinery is vital to escape adaptive immunity. In HGSOC, frequent inactivation of β2-microglobulin mutations directly leads to the loss of MHC class I molecule expression. This loss prevents cytotoxic T lymphocytes from recognizing tumor antigens (79). Under stressors such as chemotherapy, tumor cells upregulate the signal of CD47. By binding to signal regulatory protein α on the surface of macrophages, CD47 blocks their phagocytotic function. This mechanism allows tumor cells to resist the anti-tumor immune response that would normally be activated by the death of immune cell (80). The ubiquitin protein ligase E3 component N-recognin 5 regulates the p53/β-catenin pathway to promote the recruitment and activation of tumor-associated macrophages, thereby driving tumor progression (81). High expression of SGK1 and VEGFA in tumor-associated macrophages is associated with hypoxia-related immune evasion (29). These findings underscore the utility of organoids with immune-competent in identifying valuable targets to reprogram the immunosuppressive TME.

### 5.2 Evaluation of immunotherapy efficacy

Organoids are a robust preclinical model for evaluating various immunotherapies.

ICIs: ICIs counteract tumor immune evasion by blocking key inhibitory pathways (e.g., CTLA-4, PD-1/PD-L1), thereby reactivating the cytotoxic function of tumor-specific T cells (82).Co-culture systems have proven essential in predicting responses to ICIs. Wan et al. (83) demonstrated that bispecific anti-PD-1/PD-L1 antibodies could more effectively reinvigorate exhausted T cells and NK cells in HGSOC organoids than monotherapy. Furthermore, organoid platforms facilitate the stratification of patients based on immunophenotypes. Tumors with a high presence of stromal tumor-infiltrating mast cells were identified as a resistant characteristic to anti-PD-1 therapy, thus serving as a predictive biomarker (84).

Cellular Therapies: Organoids provide a 3D environment for test engineered cell therapies. Zhu et al. (85) developed ROBO1-specific CAR-NK cells that were cytotoxic against OC organoids and a safer alternative to CAR-T cells. Live-cell imaging on organoids has revealed significant heterogeneity in organoid susceptibility to NK cell-mediated killing, correlating organoid size with resistance (28).

Ex Vivo Immune Expansion: Co-culture systems are also pivotal for expanding tumor-reactive T cells. Tumor organoids can “educate” autologous peripheral blood lymphocytes to recognize and kill patient-specific tumor cells (39). This capability supports the development of adoptive cell transfer therapies using expanded autologous or healthy donor-derived T cells (86).

### 5.3 Unraveling stromal interactions and drug resistance

Beyond immune cells, the stromal competent plays a critical role in drug resistance. Complex organoid models involving cancer-associated mesenchymal stem cells and M2 macrophages have shown that they promote chemotherapy resistance and stemness through CSF-1 and Wnt/TGF-β signaling (87). M2 macrophages alone induce paclitaxel resistance, which could be reversed by TAM targets (88). Physical properties of ECM also play a role in resistance. ECM stiffening and remodeling (e.g., fibronectin upregulation) promote tumor progression (89) and drug resistance (90). Targeting endothelial signaling pathways such as Jagged1/Notch3 in organoid models has proven effective in overcoming resistance to anti-angiogenic therapy (91). These models are essential for investagating strategies that target the tumor-stroma crosstalk.

### 5.4 Emerging frontiers: novel targets and microbial interactions

**Figure 4:**
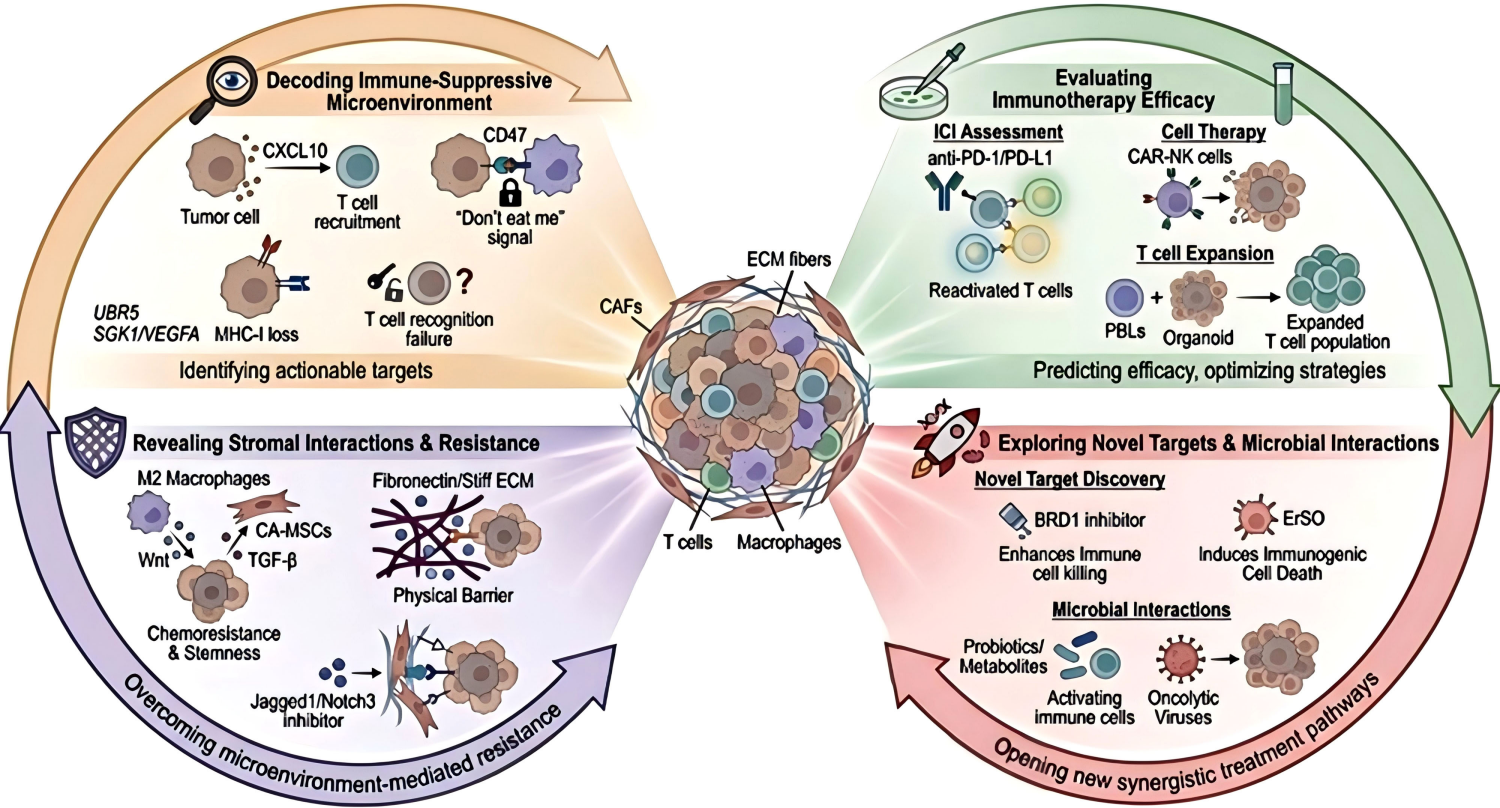
Closed-loop translational pipeline from patient to personalized therapy. This diagram summarizes how these models are used to study immune evasion, test new treatments, understand drug resistance, and explore novel therapeutic targets, accelerating the development of better therapies.

Organoid systems with immune competents facilitate the discovery of novel therapeutic targets and the exploration of new biological dimensions. High-throughput screens in organoid co-cultures have shown that BRD1 regulates T cell and NK cell cytotoxicity, suggesting BRD1 inhibitors may enhance immunotherapy (83). Organoid models have also shown that new compounds such as ErSO, which induces immunogenic cell death via the unfolded protein response (92), and traditional Chinese medicines like ZMYL that modulates immune-related pathways (93).Emerging evidence suggests that the microbiome significantly influences immunotherapy outcomes in tumor. Microbial metabolites (94) can enhance the apoptosis produced by immune checkpoint blockade. Engineered oncolytic viruses (95) can strengthen anti-tumor immunity by activating innate immune cells. To model these interactions ex vivo, technical approaches are being developed to integrate the microbiome into the organoid systems. Feasible approaches include microinjecting specific bacterial strains into the organoid lumen to mimic direct colonization (96) or using bacterial conditioned media to isolate metabolite-driven effects without infection (97). However, the major bottleneck is the ‘oxygen paradox’—sustaining anaerobic gut commensals alongside aerobic tumor cells. Emerging solutions, such as microfluidic chips with separate anaerobic-aerobic chamber are being proposed to overcome this limitation (98, 99). Integrating microbial components into organoid systems is the next frontier, promising a holistic view of the epithelial-immune-microbiome axis in OC treatment (**Figure 4**).

## 6 Limitations and prospects

The translational value of PDOs is underscored by their high predictive accuracy. De Witte et al. (100) reported an 88% concordance rate with clinical outcomes and a 100% negative predictive value, proving PDOs as a powerful tool to rule out ineffective therapies. Kopper et al. (23) confirmed that PDOs faithfully recapitulate patient responses to platinum and PARP inhibitors, validating their use in personalized treatment. While organoid models can have profound therapeutic potential in OC research, they face structural obstacles that prevent clinical translation. One main limitation is the lack of functional vascular networks, limiting organoid size and necrosis due to inadequate nutrients diffusion (101). In immuno-oncology, the absence of vasculature prevents modeling of leukocyte trafficking, which is a step where immune cells migrate from circulation into the tumor site. Without this flow of immune cells, static models may not capture the systemic responses necessary for evaluating checkpoint inhibitors or cell therapies.

The wide use of mouse-derived basement membrane extracts such as Matrigel poses a major limitation on reproducibility. Although considered the “gold standard”, its undefined and batch-variable composition introduces uncontrollable confounders that prevent standard drug screening (69). Further, the xenogeneic nature of Matrigel may induce non-specific immune activation in co-culture systems, resulting in confusing experimental results. Decellularized ECM technology has emerged as a promising alternative. By preserving the tissue-specific biochemical composition and topology of the native ovary or omentum, decellularized ECM provides physiologically relevant microenvironment (102). However its application is currently constrained by variability between donors, potential nucleic acid retention, and the loss of signaling molecules during processing.

The bio-fabrication technologies are beginning to address these architectural constraints. 3D bioprinting can be used to map cells spatially (103). This allows researchers to build complex tumor-stroma interfaces. For instance, Xu et al. printed OC cells along with fibroblasts to dissect stromal interactions (104). Although promising, this field is still in its early stage for OC because of lacking standardized bio-ink formulations. Similarity, microfluidic organ-on-a-chip platforms allow fluid flow to mimic perfusion, and partially compensating for the lack of vascularization (49). They are particularly valuable for modeling metastasis. Although not yet widely applied to OC, multi-organ chip concepts like connecting ovarian, fallopian tube, and uterine organoids (105) hold theoretical potential for modeling metastatic cascade. Heart-liver-lung chips (106) show that modeling inter-organ signaling and drug toxicity is feasible in the study the systemic immune effects of OC therapies.

Beyond technical limitations, regulatory requirements are vital for clinical use of organoids. The FDA Modernization Act 2.0 in 2022 allowed cell-based alternatives to animal testing for drug approval (107). However, this flexibility requires standards of organoid production and data integrity. For PDO-based drug sensitivity tests to serve as companion diagnostics, laboratories must comply with Clinical Laboratory Improvement Amendments requirements to ensure data integrity and minimize batch-to-batch variability, as reported in recent functional precision oncology frameworks (108). Concurrently, a precise ‘Context of Use’ is equally vital for meeting regulatory qualification, as recently emphasized by Mendes et al. (109) in their assessment of the field’s regulatory future.

In summary, the major challenge remains the faithful recapitulation of the immune microenvironment. Co-cultures have successfully included T cells and macrophages, but maintaining their stability and functional exhaustion states ex vivo is difficult. Most current media are designed to grow epithelial cells, often at the expense of immune cell viability. The transient nature of immune components may limit the predictions for long-term responses to ICIs. Finally, the microbiome is the most important but often overlooked dimension. Recent evidence suggests gut microbiota and its metabolites can modulate anti-tumor immunity (110). Current sterile organoid cultures fail to capture this “gut-ovarian axis.” Future immune-competent organoids must integrate microbiota or metabolites to provide a picture of the tumor-immune-microbiome network, thus allowing to translate preclinical results to clinical patients. Future translational efforts must therefore align with the FDA Modernization Act 2.0 by prioritizing CLIA-grade standardization and establishing precise ‘Context of Use’ to ensure these models meet the strict qualification criteria required for clinical decision-making.

## 7 Conclusion

Organoid models, especially co-culture systems with immune and stromal components, are a powerful tool to study complex TME and treatment response mechanisms in OC. They preserve patient-specific tumor heterogeneity and molecular features which provides a platform for precision medicine applications from drug screening to biomarker identification. Challenges remain in achieving standardized construction, ensuring long-term culture stability, and faithfully simulating the immune microenvironment. The convergence of multi-omics technologies is poised to address these limitations. These advancements will further empower organoid models to play a pivotal role in understanding the interactions of tumor-immune-stroma network to optimize immunotherapies and accelerate translational research. Future research should focus on developing more sophisticated organoid-TME systems.

## Glossary

OC: ovarian cancer
2D: two-dimensional
ASCs: adult stem cells
EnoC: endometrioid carcinoma
OCCC: ovarian clear cell carcinoma
iPSCs: induced pluripotent stem cells
ICIs: immune checkpoint inhibitors
HGSOC: high-grade serous ovarian carcinoma
PDX: patient-derived xenograft
ECM: extracellular matrix
PDOs: patient-derived organoids
BMP: bone morphogenetic protein
ROCK: rho-associated coiled-coil containing protein kinase
TME: tumor microenvironment
ALI: air–liquid interface
FT: fallopian tube
MC: mucinous carcinoma
OSE: ovarian surface epithelium
3D: three dimensional
PD: programmed cell death protein
IL: Interleukin
TAM: tumor-associated macrophages
PARP: poly (ADP-ribose) polymerase
FGF: fibroblast growth factor
TGF: transforming growth factor
